# Potential Anti-Atherosclerotic Properties of Astaxanthin

**DOI:** 10.3390/md14020035

**Published:** 2016-02-05

**Authors:** Yoshimi Kishimoto, Hiroshi Yoshida, Kazuo Kondo

**Affiliations:** 1 Endowed Research Department “Food for Health”, Ochanomizu University, 2-1-1 Otsuka, Bunkyo-ku, Tokyo 112-8610, Japan; kishimoto.yoshimi@ocha.ac.jp (Y.K.); kondo.kazuo@ocha.ac.jp (K.K.); 2 Department of Laboratory Medicine, Jikei University Kashiwa Hospital, 163-1 Kashiwashita, Kashiwa, Chiba 277-8567, Japan; 3 Institute of Life Innovation Studies, Toyo University, 1-1-1 Izumino, Itakura-machi, Ora-gun, Gunma 374-0193, Japan

**Keywords:** astaxanthin, oxidative stress, inflammation, lipid metabolism, glucose metabolism, atherosclerosis, cardiovascular disease

## Abstract

Astaxanthin is a naturally occurring red carotenoid pigment classified as a xanthophyll, found in microalgae and seafood such as salmon, trout, and shrimp. This review focuses on astaxanthin as a bioactive compound and outlines the evidence associated with its potential role in the prevention of atherosclerosis. Astaxanthin has a unique molecular structure that is responsible for its powerful antioxidant activities by quenching singlet oxygen and scavenging free radicals. Astaxanthin has been reported to inhibit low-density lipoprotein (LDL) oxidation and to increase high-density lipoprotein (HDL)-cholesterol and adiponectin levels in clinical studies. Accumulating evidence suggests that astaxanthin could exert preventive actions against atherosclerotic cardiovascular disease (CVD) via its potential to improve oxidative stress, inflammation, lipid metabolism, and glucose metabolism. In addition to identifying mechanisms of astaxanthin bioactivity by basic research, much more epidemiological and clinical evidence linking reduced CVD risk with dietary astaxanthin intake is needed.

## 1. Introduction

Astaxanthin (3,3′-dihydroxy-β,β′-carotene-4,4′-dione), a red carotenoid pigment classified as a xanthophyll, is known to have a powerful antioxidant ability. Oxidative stress and inflammation are involved in the development of atherosclerotic diseases, and therefore much attention has been paid to antioxidant foods as potential agents for preventing or treating these diseases. Astaxanthin is one of the promising agents in the prevention of oxidative stress-related diseases, and both the basic and clinical research on the health benefits of astaxanthin has quickly developed over the past few years.

Cardiovascular disease (CVD) is the leading cause of death worldwide. The coexistence of dyslipidemia, impaired glucose tolerance, and hypertension with accumulated visceral fat has been termed metabolic syndrome, which increases synergistically the risk of CVD. Metabolic syndrome is often characterized by oxidative stress, a disturbance in the balance between the production of reactive oxygen species (ROS) and antioxidant defenses. It has recently become clear that the effects of astaxanthin go beyond antioxidant properties. Accumulating evidence suggests that astaxanthin could exert cardioprotective actions by improving oxidative stress, inflammation, lipid metabolism, and glucose metabolism. The objective of this review is to summarize the findings regarding the bio-functions of astaxanthin in the prevention of atherosclerosis.

## 2. Food Sources and Bioavailability of Astaxanthin

Astaxanthin is biosynthesized by microalgae, bacteria, and fungi, and concentrates higher up the food chain. Humans commonly consume astaxanthin from seafood such as salmon, trout, shrimp, lobster, crab, and fish eggs. Astaxanthin is also fed to farmed seafood to add red color. The content of astaxanthin was reported as 6–8 mg/kg flesh in farmed Atlantic salmon, and 6 mg/kg flesh and 25 mg/kg flesh in large trout in the European and Japanese markets, respectively [1]. The highest known level of astaxanthin in nature is in the chlorophyte alga *Haematococcus pluvialis*, which has become a primary source of the astaxanthin used in the food industry [2,3]. Not only the content but also the composition of isomers of astaxanthin differ among organisms. *H. pluvialis* produces the all-*trans* geometric form 3S, 3′taxanthin, and therefore this type is most largely ingested by humans [1]. Human cannot synthesize astaxanthin, and the ingested astaxanthin cannot be converted to vitamin A; excessive intake of astaxanthin will thus not cause hypervitaminosis A [4,5]. In 1987, the U.S. Food and Drug Administration (FDA) authorized astaxanthin as a feed additive for the aquaculture industry, and in 1999 the FDA approved astaxanthin as a dietary supplement [6]. The use of astaxanthin as a dietary supplement has been rapidly growing in many countries. Japan is one of the global pioneers in astaxanthin research and production. The FDA first awarded the “generally recognized as safe” (GRAS) status to astaxanthin extracted from *H. pluvialis* produced by a Japanese company in 2010.

Human clinical studies have used orally administered astaxanthin in a dose ranging from 4 mg to 100 mg/day [5]. In experimental and human studies, astaxanthin seems to be well tolerated, and no notable toxicity has been described. In a study by Coral-Hinostroza *et al.*, male subjects ingested a single meal containing a 10 mg dose equivalent of astaxanthin from astaxanthin diesters and then, after four weeks, given a dose of 100 mg astaxanthin equivalents. A non-linear dose–response relationship and selective absorption of *Z*-isomers were observed, and the plasma elimination half-life was estimated as 52 ± 40 h [7]. The presence of dietary fat enhances the assimilation of astaxanthin in the small intestine [8]. It is also noteworthy that the bioavailability of astaxanthin is decreased in smokers by approximately 40% [8].

## 3. Multiple Anti-Atherosclerotic Effects of Astaxanthin

### 3.1. Anti-Oxidation

**Figure 1:**
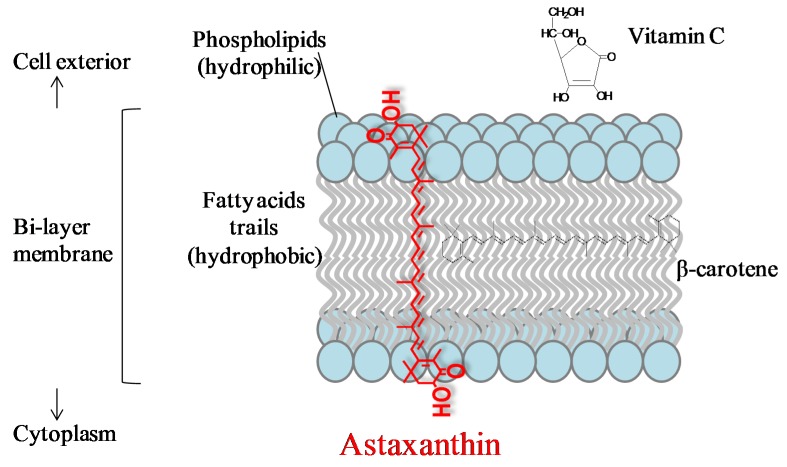
Transmembrane orientation of astaxanthin [1,9].

Carotenoids contain long conjugated double bonds in a polyene chain that are responsible for antioxidant activities by quenching singlet oxygen and scavenging radicals to terminate the chain reaction. Astaxanthin contains a conjugated polyene chain at the center and hydroxy and keto moieties on each ionone ring. Owing to its unique molecular structure, astaxanthin shows better biological activity than other antioxidants, because it can link with the cell membrane from the inside to the outside [1,9] (Figure 1). The polyene chain in astaxanthin traps radicals in the cell membrane, while the terminal ring of astaxanthin can scavenge radicals both at the surface and in the interior of the cell membrane [10].

Astaxanthin is reported to be more effective than β-carotene in preventing lipid peroxidation in solution [11] and various biomembrane models such as egg yolk phosphatidylcholine liposomes [12] and rat liver microsomes [13]. Goto *et al.* reported in 2001 that astaxanthin was approximately twofold more effective than β-carotene in the inhibition of liposome peroxidation induced by ADP and Fe^2+^ [10]. Their report was the first to demonstrate that astaxanthin could trap radicals not only at the conjugated polyene chain but also in the terminal ring moiety. The proposed molecular interaction was as follows: (1) the two terminal rings interact with the hydrophilic polar site of membrane phospholipids; and (2) the hydroxyl and carbonyl groups form an intramolecular hydrogen-bonded five-membered ring, increasing the hydrophobicity of astaxanthin. It is well known that the activity of carotenoids can be shifted from antioxidant to pro-oxidant according to their concentrations, high oxygen tension, or interactions with other co-antioxidants [14]. Martin *et al.* divided 17 carotenoids into three classes: (1) those without significant antioxidative properties; (2) those with good antioxidative but also pro-oxidative properties; and (3) those with strong antioxidative and without any pro-oxidative properties. Astaxanthin was categorized as class (3), whereas β-carotene and lycopene were identified as class (2) [15].

The increase in the susceptibility of low-density lipoprotein (LDL) and cell membrane lipids to oxidative processes contributes to atherosclerosis and thrombus formation. Our group was the first to report that astaxanthin protected human LDL against oxidative attack [16]. Compared to α-tocopherol and lutein, astaxanthin showed a greater antioxidative effect on LDL oxidation induced by AMVN-CH_3_O (2,2-azobis-4-methoxy-2,4-dimethylvaleronitrile) *in vitro*. To confirm the antioxidant effect of astaxanthin *ex vivo*, we recruited 24 healthy adults to consume astaxanthin purified from krill at 0, 1.8, 3.6, 14.4, and 21.6 mg/day for 14 days and then measured the changes in LDL oxidizability. At the end of the study, astaxanthin consumption significantly prolonged the lag time, a marker of the susceptibility of LDL to oxidation, at the dose levels of 3.6 mg/day and higher. Importantly, an intake of 3.6 mg astaxanthin is equivalent to approximately 165 g of salmon flesh. Nakagawa *et al.* reported the efficacy of astaxanthin supplementation (6 and 12 mg/day) on phospholipid hydroperoxides (PLOOH) levels in erythrocytes in 30 healthy subjects. After 12 weeks of administration, decreased PLOOH levels and increased astaxanthin in erythrocytes were observed [17]. Karppi *et al.* also reported that supplementation of astaxanthin for 12 weeks reduced the revels of plasma 12- and 15-hydroxy fatty acids in healthy males [18]. In obese and overweight adults, supplemental astaxanthin (5 and 20 mg/day) reduced biomarkers of oxidative stress including malondialdehyde (MDA) and isoprostane, and increased superoxide dismutase (SOD) and total antioxidant capacity (TAC) [19]. These findings suggest that astaxanthin may decrease *in vivo* lipid peroxidation.

There are several antioxidant enzymes that catalyze reactions to counteract free radicals and ROS. Nuclear factor erythroid-related factor 2 (Nrf2) is a master regulator of the antioxidant response and xenobiotic metabolism through the regulation of a wide range of antioxidant and Phase II detoxification genes [20,21]. Tripathi *et al.* demonstrated that astaxanthin treatment attenuated cyclophosphamide-induced oxidative stress, DNA damage, and cell death in rat hepatocytes through an Nrf2-antioxidant response element (ARE) pathway [22]. It was further observed that the levels of Nrf2 and the targeted phase-II enzymes, *i.e.*, NAD(P)H dehydrogenase, quinone 1 (NQO1) and heme oxygenase 1 (HO-1) were increased with astaxanthin treatment. Interestingly, astaxanthin showed synergistic effects on the induction of the cellular glutathione level and the mRNA expression of Nrf2 and its target genes (NQO1, HO-1, and glutathione *S*-transferase mu 2 [GSTM2]) when combined with docosahexaenoic acid (DHA) or eicosapentaenoic acid (EPA) in a human hepatoma cell line [23].

Paraoxonase 1 (PON1), which is mainly responsible for the breakdown of lipid peroxides before they can accumulate in LDL, is an enzyme located on circulating high-density lipoprotein (HDL) particles, but the enzymatic activity of PON1 is readily inactivated by oxidants [24]. In hypercholesterolemic rabbits, astaxanthin prevented protein oxidation and changes in PON1 and thioredoxin reductase (TrxR-1) activities [25]. TrxR-1 is a redox-active protein that efficiently regenerates oxidized thioredoxin. These effects were not related to a direct effect of astaxanthin on these enzymes, because astaxanthin enhanced TrxR-1 and had no effect on PON1 activity *in vitro* [25]. It was reported that regular physical activity might increase PON1 activity [26]. Astaxanthin supplementation (4 mg/day) for 90 days showed a beneficial effect in improving PON1 activity as well as the total sulphydryl group content in young soccer players [27]. The same researchers also reported that post-exercise creatine kinase (CK) and aspartate aminotransferase (AST) levels were significantly lower in their astaxanthin group compared to a placebo group [28]. Astaxanthin might be of special interest for athletes who are more susceptible to oxidative stress.

### 3.2. Anti-Inflammation

**Figure 2:**
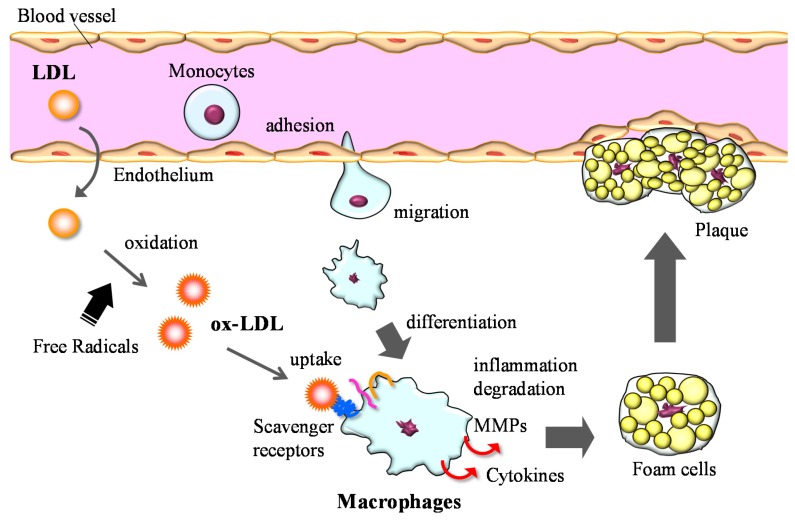
Role of macrophages in the development of atherosclerosis.

Chronic inflammation is the main pathophysiological factor in many diseases, such as diabetes, hypertension and atherosclerosis. Inhibiting the production of intracellular ROS is a general way to suppress the pro-inflammatory signals, and thus modulators of redox balance are considered the key regulators of inflammatory responses. Macrophages play a central role in inflammation and atherosclerosis progression (Figure 2). The scavenger receptor-mediated uptake of oxidized-LDL by macrophages leads to the formation of foam cells and the development of atherosclerotic plaque. The class A scavenger receptor (SR-A) and CD36 are responsible for the major part of oxidized LDL uptake by macrophages, suggesting pro-atherogenic roles of SR-A and CD36 [29,30]. Additionally, in inflammation states, macrophages produce excess amounts of matrix-degrading enzymes, pro-inflammatory cytokines/chemokines, nitric oxide (NO), and cyclooxygenase-2 (COX-2) [31]. Matrix metalloproteinases (MMPs), a family of Zn^2+^-dependent endopeptidases, are responsible for the degradation of most extracellular matrix proteins, and they mediate tissue remodeling in various pathologic conditions [32]. The expression of MMPs is increased in atherosclerotic lesions and is linked to weakening of the vascular wall due to degradation of the extracellular matrix [33,34,35].

In our previous study, astaxanthin remarkably suppressed the expression of the scavenger receptors SR-A and CD36) the activity and the expression of MMPs, and the mRNA expression of various inflammatory mediators, *i.e.*, tumor necrosis factor (TNF)-α, interleukin (IL)-1β, IL-6, inducible nitric oxide synthase (iNOS), and COX-2, in THP-1 macrophages [36]. We speculated that the antioxidant property of astaxanthin might account for its inhibitory effects on macrophage inflammation through the inhibition of nuclear factor-kappa B (NF-κB) activation. The pro-inflammatory cytokines, prostaglandins, and NO produced by activated macrophages play critical roles in atherogenesis. The inhibition of pro-inflammatory cytokine secretion from macrophages might be one of the mechanisms mediating the beneficial effects of antioxidants on atherosclerosis development. Another study reported that astaxanthin decreased the expression of pro-inflammatory mediators such as prostaglandin E2, TNF-α, and IL-1β by suppressing IκB-dependent NF-κB activation in both lipopolysaccharide (LPS)-stimulated RAW264.7 cells and primary macrophages [37].

Macedo *et al.* showed that astaxanthin significantly reduced the production of pro-inflammatory cytokines (TNF-α and IL-6) in LPS-stimulated neutrophils [38]. Their study also revealed that astaxanthin enhanced neutrophil phagocytic and microbicidal capacity and suppressed superoxide anion and hydrogen peroxide production, which might be mediated by calcium released from intracellular storage and NO production [38]. These studies indicated that the anti-inflammatory effects of astaxanthin through its suppression of NF-κB activation may be based on its antioxidant activity. In addition, astaxanthin treatment reduced the secretion of pro-inflammatory cytokines (IL-1β, IL-6 and TNF-α) in H_2_O_2_-stimulated U937 mononuclear cells, and this property was elicited by a restoration of the basal SHP-1 protein expression level and reduced NF-κB (p65) nuclear expression [39]. SHP-1 is a protein tyrosine phosphatase that acts as a negative regulator of inflammatory cytokine signaling, and SHP-1 deficiency in mice causes spontaneous inflammation and autoimmunity [40]; the authors of that study proposed that astaxanthin most likely inhibits the ROS-induced production of NF-κB transcription factor through the restoration of physiological levels of SHP-1 [39].

Ischemia-reperfusion (IR) is a complex inflammatory process that includes major oxidative stress induced by ischemia and hypoxia [41]. Curek *et al.* found that astaxanthin treatment significantly decreased the conversion of xanthine dehydrogenase (XDH) to xanthine oxidase (XO), which reduced the level of oxidative stress in hepatocellular injury following IR in rats [42]. Another study showed that apoptosis and autophagy caused by hepatic IR injury were attenuated by astaxanthin pretreatment following a reduction in the release of ROS and inflammatory cytokines via the mitogen-activated protein kinase (MAPK) pathway in mice [43]. With respect to IR, some studies reported the protective effects of astaxanthin on brain and myocardial injury following IR [44,45,46].

In a clinical study, Park *et al.* studied the possible immune-enhancing, anti-oxidative, and anti-inflammatory activity of astaxanthin in healthy adult females, and their results showed that astaxanthin supplementation (2 and 8 mg/day) for eight weeks could decrease both the level of a DNA oxidative damage biomarker and inflammation, and enhance the immune response [47]. The enhancement of the immune response was also observed in dogs fed astaxanthin [48], β-carotene [49], and lutein [50].

### 3.3. Lipid Metabolism

Astaxanthin has been reported to improve dyslipidemia and metabolic syndrome in animal models [51,52,53]. In apoE knockout mice fed a high-fat and high-cholesterol diet, astaxanthin increased the levels of LDL receptor (LDLR), 3-hydroxy-3-methylglutaryl CoA (HMG-CoA) reductase and sterol regulatory element binding protein 2 (SREBP-2) in the liver, which might be responsible for the hypocholesterolemic effect of astaxanthin [53]. In the same experiment, the expressions of carnitine palmitoyl transferase 1 (CPT1), acetyl-CoA carboxylase β (ACACB) and acyl-CoA oxidase (ACOX) mRNA were significantly increased by astaxanthin supplementation, suggesting that the triglyceride-lowering effect of astaxanthin might be due to increased fatty acid β-oxidation in the liver [53]. Iizuka *et al.* investigated the effects of astaxanthin on key molecules in cholesterol efflux from macrophages. Their study revealed that astaxanthin did not modify peroxisome proliferator-activated receptor (PPAR)-γ or liver X receptor (LXR)-α and -β levels, but it increased the expression of ATP-binding cassette transporters (ABC) A1 and G1, thereby enhancing the cholesterol efflux from macrophages [54]. In diet-induced obesity in mice, astaxanthin significantly lowered the plasma triglyceride, alanine transaminase (ALT) and AST levels and increased the mRNA expression of antioxidant genes regulated by Nrf2 in the liver [55]. In addition, astaxanthin decreased macrophage infiltration and apoptosis of vascular cells in atherosclerotic plaques and provided stabilization of the plaques in hyperlipidemic rabbits [56].

To determine the lipid metabolism-modulating effect of astaxanthin in humans, we conducted a placebo-controlled study of astaxanthin administration at doses of 0, 6, 12, and 18 mg/day for 12 weeks with 61 non-obese subjects with mild hypertriglycemia. Multiple comparison tests showed that 12 and 18 mg/day of astaxanthin significantly reduced the subjects’ triglyceride levels, and the 6- and 12-mg doses significantly increased HDL-cholesterol. The serum adiponectin level was also increased by astaxanthin (12 or 18 mg/day), and the changes of adiponectin positively correlated with the HDL-cholesterol changes [57]. The HDL-increasing effect of astaxanthin is thus of significant interest, because a very limited number of dietary factors were suggested to increase HDL-cholesterol concentrations [58]. Our study showed a markedly positive correlation between the percentage change of adiponectin and that of the HDL-cholesterol level.

Although the mechanisms of astaxanthin-mediated adiponectin elevation are still poorly understood, several investigations have shown that there is a significant and independent association between serum adiponectin and HDL-cholesterol [59], and that adiponectin may directly regulate HDL metabolism through a dual effect on the very-low-density lipoprotein (VLDL)-triglyceride pool and hepatic lipase [60,61,62]. Another mechanism of the astaxanthin-induced increase in HDL-cholesterol may be considered, albeit by an implicit action, due to the increased ABCA1 expression and cholesterol efflux from macrophages through the actions of adiponectin increased by astaxanthin [54,63]. In contrast, a recent meta-analysis of seven randomized controlled trials (including our trial [57]) failed to identify a significant effect of astaxanthin on the plasma lipid profile, but a slight glucose-lowering effect was observed [64]. The review’s authors mentioned that the study interpretation had limitations regarding the heterogeneous populations, the varying concepts of the studies, and the different quantities of astaxanthin used.

For the improvement of astaxanthin’s oral bioavailability, a novel prodrug of astaxanthin (CDX-085) was developed (approximately 10-fold more potent compared to pure astaxanthin). The oral administration of CDX-085 effectively lowered the total cholesterol and aortic arch atherosclerosis in LDL receptor-deficient mice and the triglyceride levels in ApoE-deficient mice [65]. Khan *et al.* reported that CDX-085 reduced thrombi and increased blood flow in a mouse model of oxidative stress-induced thrombus, which appeared to be partially mediated by increased NO and decreased peroxynitrite in endothelial cells and platelets [66].

Several studies have indicated that changes in the intracellular redox balance can modify lipid metabolism. Indeed, oxidative stress was associated with lipid accumulation in adipose tissue [67,68] and affected the regulation of hepatic lipid synthesis [69]. During exercise, ROS generation in skeletal muscle increases along with the elevation of energy expenditure, and such ROS may also affect the utilization of energy substrates in muscle, which leads to a disorder in the lipid metabolism. Aoi *et al.* reported that astaxanthin accelerated lipid utilization during exercise, leading to improved endurance and an efficient reduction of body fat with training in mice [70]. An increase of fatty acyl-CoA uptake into the mitochondria via CPT1 during exercise may be involved in the promotion of lipid metabolism by the antioxidant activity of astaxanthin. According to these findings, astaxanthin is expected to improve aerobic performance and body weight control by the modification of muscle energy metabolism via its antioxidant effect.

### 3.4. Glucose Metabolism and Blood Pressure Control

Growing evidence suggests that diabetes and other disorders of glucose metabolism, such as impaired glucose tolerance (IGT), needs to be taken into consideration as independent risk factors for CVD [71,72,73]. Since oxidative stress promotes insulin resistance in obesity and type 2 diabetes, it is crucial to find effective antioxidants for decreasing this threat. As mentioned above, a recent meta-analysis of randomized controlled trials showed that astaxanthin supplementation slightly lowered glucose levels [64]. Antidiabetic effects of astaxanthin could be explained by means of several proposed mechanisms. In db/db mice, astaxanthin showed a protective effect against oxidative stress and cytotoxicity in pancreatic β-cells [74]. Arunkumar *et al.* reported that astaxanthin activated the hepatic IRS-PI3K-Akt signaling pathway and improved glucose metabolism in liver of high-fructose and high-fat diet (HFFD)-fed mice [75]. Another study reported that astaxanthin treatment normalized the activities of hexokinase, pyruvate kinase, glucose-6-phosphatase, fructose-1,6-bisphosphatase and glycogen phosphorylase and increased the glycogen reserves in the liver of HFFD-fed mice [76]. In addition, astaxanthin decreased the HFFD-induced activation of serine kinases (JNK and ERK). The anti-obesity effect of astaxanthin has been reported in high fat-fed mice; astaxanthin was shown to increase fatty acid utilization [52], which can be responsible for its anti-diabetic effect. It is known that fucoxanthin, a xanthophyll carotenoid present in brown seaweeds, induces uncoupling protein 1 (UCP1) in mitochondria, leading to the oxidation of fatty acids and heat production in white adipose tissue [77]. Fucoxanthin supplementation was also tested in humans: a 16 week supplementation with 4 mg/day promoted weight loss, reduced body and liver fat content, and improved liver function tests in obese non-diabetic women [78]. The mechanisms of anti-diabetic and anti-obesity effect of astaxanthin remain unclear and has yet to be studied in clinical condition.

Studies in animal models of insulin resistance and fatty liver have demonstrated that hepatic steatosis and endoplasmic reticulum (ER) stress are linked to each other [79]. A recent study showed that disruption of ER homeostasis led to chronic unfolded protein response (UPR) and induced inflammation and insulin resistance in the liver [80]. Hepatic ER stress can promote *de novo* lipogenesis, while lipids can exacerbate ER stress, a situation that creates a vicious cycle. Bhuvaneswari *et al.* reported that astaxanthin reduced hepatic ER stress, ROS production, phosphorylation of JNK, and NF-κB-mediated inflammation in HFFD-fed mice [81]. Astaxanthin may also have prevented the progression of diabetic nephropathy by decreasing renal oxidative stress and by preventing renal cell damage in db/db mice [82]. Another study reported that astaxanthin beneficially affected both sucrose-induced elevations of blood pressure and insulin resistance at relatively high doses in rats [83]. Astaxanthin may have an innate antihypertensive effect, because astaxanthin administration lowered the blood pressure and delayed the incidence of stroke in spontaneously hypertensive rats (SHRs) [84,85]; however, this effect is not well-defined, and further studies to elucidate the antihypertensive effect of astaxanthin should be performed.

Diabetes-induced cognitive deficit is a prevalent disease with substantial morbidity and mortality, presenting a global health problem with serious economic burdens. Xu *et al.* performed Morris water maze tests to test whether astaxanthin would affect the cognitive function of diabetic rats, and their findings demonstrated that astaxanthin improved the escape latency, mean path length, mean percentage of time spent in the target quadrant, and the number of times of crossing platform [86]. Xu *et al.* also demonstrated that astaxanthin ameliorated the caspase-3/9 expression and promoted the expression of PI3K/Akt in the rat cerebral cortex and hippocampus [86]. Importantly, Japanese researchers demonstrated that astaxanthin-rich *H. pluvialis* extract (6 mg or 12 mg/day for 12 weeks) improved cognitive function in healthy aged humans [87].

## 4. Conclusions

Beyond the antioxidant ability of astaxanthin, many studies have established that astaxanthin can exert preventive actions against atherosclerosis via its potential to improve inflammation, lipid metabolism, and glucose metabolism. Table 1 shows the summary of above-mentioned investigations into the anti-atherosclerotic effects of astaxanthin.

The current data may be promising in clinical conditions, but the therapeutic potential of this natural compound in humans remains to be established. In addition to identifying the mechanisms underlying astaxanthin’s bioactivity by basic research, much more epidemiological and clinical evidence is needed. This review provides new insight into the use of astaxanthin as a preventive or therapeutic strategy for atherosclerotic diseases.

**Table 1:** Clinical studies and a meta-analysis investigating the potential anti-atherosclerotic effects of astaxanthin.

**Anti-Oxidation**			
Iwamoto *et al.* (2000) [16]	Healthy volunteers (*n* = 24)	Open labeled; 2 weeks; 1.8, 3.6, 14.4 or 21.6 mg/day	↓ LDL oxidation
Nakagawa *et al.* (2011) [17]	Middle-aged and senior subjects (*n* = 30)	Randomized, double-blind, placebo controlled; 12 weeks; 6 or 12 mg/day	↓ phospholipid peroxidation in erythrocytes
Karppi *et al.* (2007) [18]	Healthy non-smoking males (*n* = 40)	Randomized, double-blind, placebo controlled; 12 weeks; 8 mg/day	↓ plasma 12- and 15-hydroxy fatty acids
Choi *et al.* (2011) [19]	Obese and overweight adults (*n* = 23)	Randomized, double-blind; 3 weeks; 5 or 20 mg/day	↓ plasma MDA, isoprastane ↑ SOD, TAC
**Anti-Inflammation**			
Park *et al.* (2010) [47]	Healthy female college students (*n* = 42)	Randomized, double-blind, placebo controlled; 8 weeks; 0, 2 or 8 mg/day	↓ plasma 8-hydroxy-2′-deoxyguanosine, CRP
**Lipid Metabolism-Modulating**			
Yoshida *et al.* (2010) [57]	Non-obese subjects with mild hypertriglycemia (*n* = 61)	Randomized, placebo-controlled study; 12 weeks; 0, 6, 12 or 18 mg/day	↓ serum TG, ↑ HDL-C, adiponectin
Ursoniu *et al.* (2015) [64]		Meta-analysis of seven randomized controlled studies	No significant effect on plasma lipid profile (LDL-C, HDL-C, TG)
**Glucose Lowering**			
Ursoniu *et al.* (2015) [64]		Meta-analysis of seven randomized controlled studies	Slight lowering effect on plasma glucose
